# The health impact of climate change on the women in reproductive age: a study of coastal communities in Satkhira, Bangladesh

**DOI:** 10.3389/fpubh.2025.1560498

**Published:** 2025-06-24

**Authors:** Md. Noman Amin, Md. Alamgir Hossain, Md. Rakibul Islam, Sabuj Mondal, Md. Hossain Ali

**Affiliations:** ^1^Department of Sociology, Gopalganj Science and Technology University, Gopalganj, Bangladesh; ^2^Department of Sociology, Rabindra University, Bangladesh, Dhaka, Bangladesh; ^3^Research and Integrated Thoughts Ltd., Dhaka, Bangladesh

**Keywords:** climate change, women health, reproductive health, physical and psychological health, coastal communities

## Abstract

**Background:**

Climate change and health issues pose a global threat, particularly in developing countries like Bangladesh. Within the socio-economic structure in coastal regions, women played a crucial role in contributing livelihood and living resources, while new climatic ecology creates chaotic relationships between environment and human health. The emerging adverse climatic ecology is directly and indirectly affecting them in the sphere of their both outdoor and indoor activism. This study explores the health impacts of climate change on the women of reproductive age (ages between 14 and 49 from the Bangladeshi perspective) living in coastal communities, i.e., Satkhira, Bangladesh.

**Methods:**

To choose study locations, this study conducted a literature survey to find out the most vulnerable coastal region of Bangladesh. The study has selected the five most vulnerable unions of Shyamnagar upazila in Satkhira district. This study adopted a multi-method approach combining in-depth interviews and KIIs. Based on this methodological guide, this study interviewed 25 women and 5 married men, while their responses have further been supplemented and validated by KIIs with health workers and medical officers.

**Results:**

Findings show that climate change posture to new climatic ecology facilitating adverse situations that lead to the intrusion of saline water within communities, lack of fresh and drinkable water, women exposure to waterborne diseases resulting from both salinity and scarcity of fresh usable water, skin disorders, gynaecological and reproductive illnesses, and vector-borne diseases. Women also bear an encountered burden in their struggle to acquire water and good health, including limited hygiene facilities and maternal care. This dilemma is even worsened during the summer season, which exposes women to heat waves, resulting in physical complications such as anaemia, pregnancy risks, heat stroke, dehydration, hypertension and psychological complications like anxiety, stress and depression.

**Conclusion:**

Breaches in awareness and prevention practices were outlined from the study, as there is a need to realize integrated solutions to address the environmental and health challenges of the populations. Further, there is an absolute need to continue improving access to safe water, healthcare services, and education as a way to build resilience in affected communities.

## 1 Introduction

The intersection of climate change and health presents a pressing global challenge, disproportionately affecting vulnerable populations, particularly in developing regions. The Satkhira district of Bangladesh exemplifies these challenges, grappling with the health impacts of rising sea levels, salinity intrusion, and extreme weather events. Studies have documented the historical rise in sea levels in Satkhira, emphasizing its tangible consequences for coastal communities (1). Livelihood-dependent households in Satkhira have experienced significant challenges due to salinity intrusion. Research has shed light on white spot disease (WSD) in farmed shrimp in Satkhira, underlining the environmental and economic vulnerabilities faced by local industries (2). Studies have also explored how these households adapt to changing conditions, highlighting sector-specific strategies for resilience (3). Broader research on climate change adaptation in Bangladesh’s coastal regions, including Satkhira, underscores the importance of targeted efforts to address vulnerabilities (4) (Table 1).

**Table 1:** Women exposure to climate induced health risk (26).

Climate-sensitive diagnosis (ICD-10 group)	Share of all cases	Total cases 2017–22	Estimated female cases*
Diarrhea & infectious gastro-enteritis	28.5%	≈ 817,000	≈ 456,000
Pneumonia (viral, bacterial & unspecified)	18.9%	≈ 541,000	≈ 302,000
Anxiety, panic & other anxiety disorders	13.2%	≈ 378,000	≈ 211,000
Urinary-tract infection	7.9%	≈ 226,000	≈ 126,000
Cholera	3.0%	≈ 86,800	≈ 48,000
Typhoid fever	3.3%	≈ 93,700	≈ 52,000

*Female numbers assume the overall 55.82% female share reported in the dataset.

Climate stressors, such as extreme heat and water salinity, exacerbate maternal health risks, increasing instances of preterm births, low birth weight, hypertension, and mental health issues like anxiety and depression (5). Menstrual health and hygiene management (MHM) are increasingly recognized as critical issues intersecting with climate change. Water scarcity and inadequate sanitation infrastructure in climate-affected regions like Satkhira hinder menstrual hygiene management, exposing adolescent girls to infections and other health complications. Climate-induced displacement further disrupts access to essential sanitary products and safe spaces for MHM, with significant implications for physical and mental health (6) (Table 2).

**Table 2:** Climate induced health problems of women.

Condition / risk	Key statistic for women	Climate driver
Hypertension (general adult)	31.8% prevalence among coastal adults; strongly associated with high-salinity drinking-water exposure (≥ 600 mg Na/L) (27)	Chronic seawater intrusion
Pregnancy-related hypertension / pre-eclampsia	9.28% of hospital deliveries showed hypertensive disorders in a coastal cohort; risk peaks in the dry (saltiest) season (28)	Sodium-rich groundwater & pond water
Kidney disease (CKD)	Rising CKD clusters reported among coastal women forced to drink saline water (29)	Long-term salt and heavy-metal co-exposure
Vector-borne dengue	During the 2019 mega-outbreak, women, children and older adults were the most frequently hospitalized groups, driven by warmer, wetter monsoon seasons (30)	Temperature, rainfall
Mental-health stress	Anxiety disorders now make up 13% of all climate-linked visits; women report sleeplessness over water collection loans and heat stress (26)	Heat waves, economic shocks
Pregnancy complications linked to salinity	High sodium intake from tube-well water raises risk of pre-eclampsia, organ damage and maternal death (29)	Sea-level rise, cyclone-driven saltwater flooding

**Figure 1:**
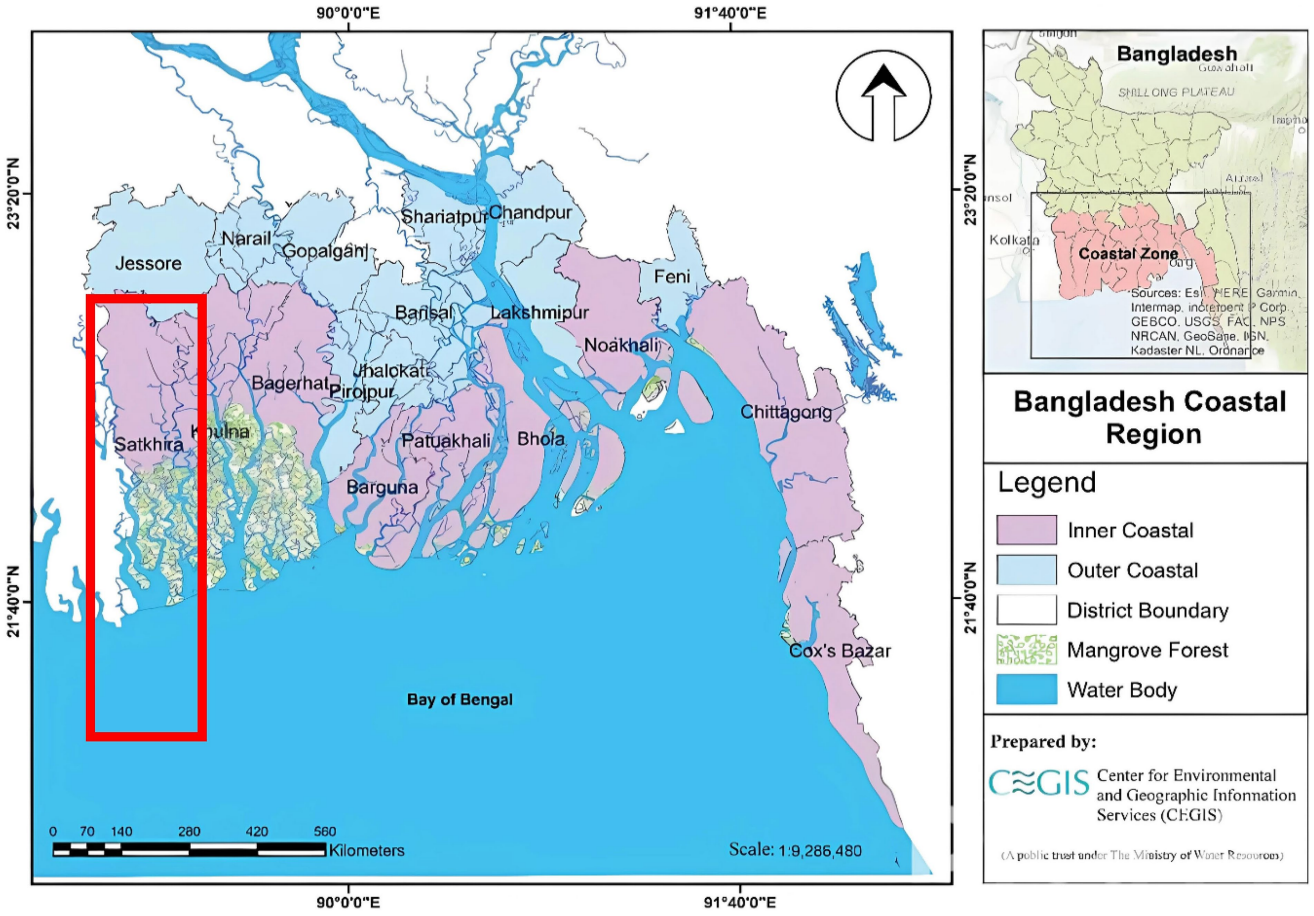
Coastal region of Bangladesh and the location of Satkhira District (12).

Adaptation strategies tailored to unique needs have been identified as crucial for mitigating the adverse effects of climate change (7). Research on the effects of climate-induced stressors on fishing communities and riverine flooding has revealed significant health risks (8). Additionally, gender-sensitive approaches to climate change adaptation are critical, as women in Satkhira face unique challenges, including stunting and malnutrition among adolescent mothers (9) and limited access to maternal health services (10) (Figure 1).

About 30% of the total population resides within the coastal region of Bangladesh (10). Initiatives focusing on water resource management, sanitation, and hygiene education are crucial for mitigating health impacts from extreme weather and salinity intrusion (5). Healthcare providers also require improved training to address emerging climate-related health issues, including vector-borne diseases, heat-related illnesses, and pregnancy complications. Integrating climate change into medical education and professional development is essential for equipping providers to handle these challenges (11). Effective policy development and intervention plans depend on a knowledge of how climate change affects health outcomes. But in low- and middle-income countries, like Bangladesh, the actual scope of these consequences is still unknown because of data shortfall. This study aims to explore climate- induced health complexities faced by the women in reproductive ages and identify common health issues during pregnancy. This study also explores the mental health among women induced due to climate change, and explore the engagement of local stakeholders in addressing climate-related health challenges affecting women and children.

## 2 Methodology

**Figure 2:**
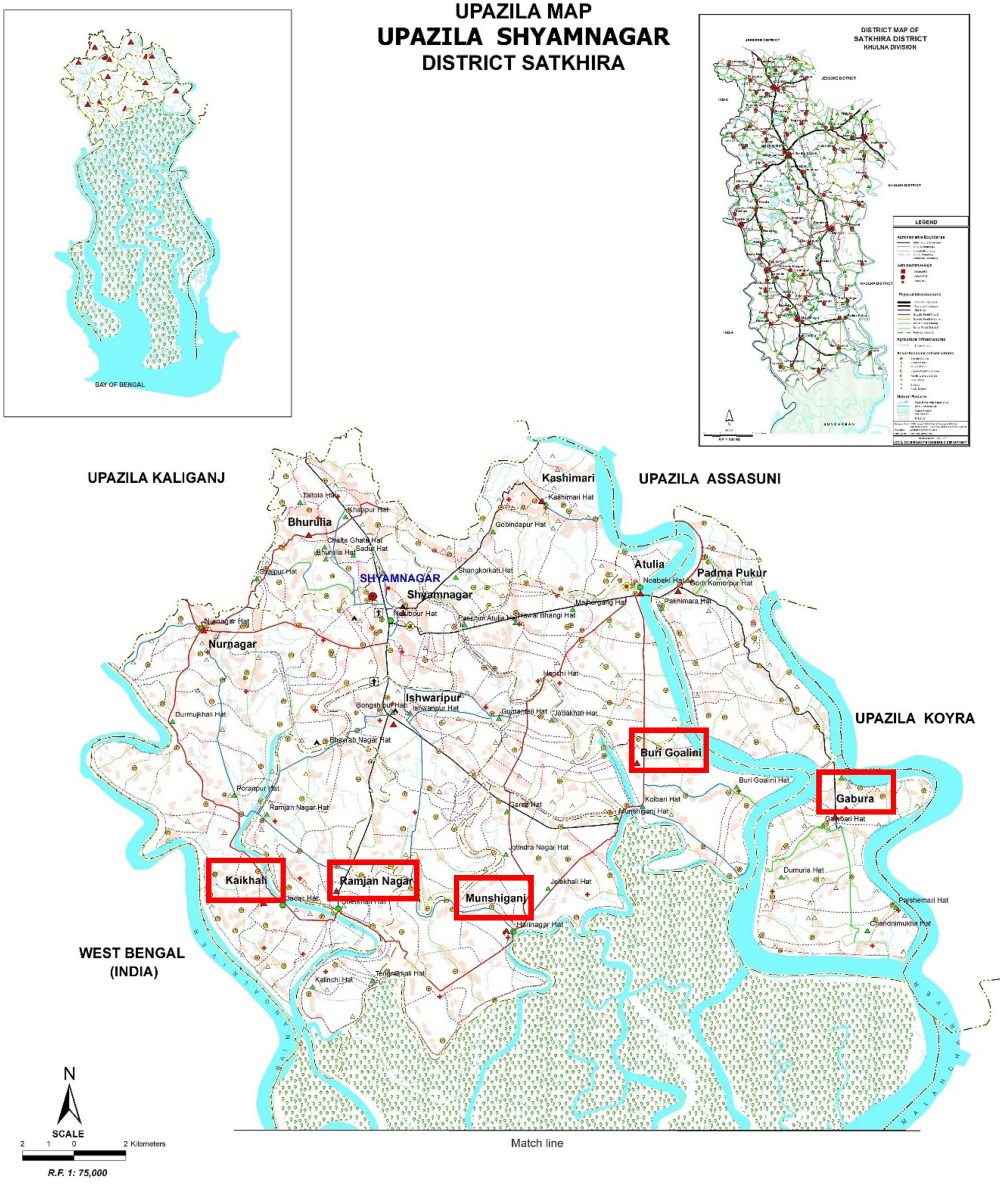
Map of Shyamnagar upazilla and footprint of the study area (12).

The coastal region of Bangladesh, i.e., Satkhira, Khulna, Bagerhat, Pirojpur, Patuakhali, Bhola, Noakhali, Chittagong, and Cox’s Bazar (12) is mostly associated with climate-induced vulnerabilities. Among them, the Shyamnagar upazilla of Satkhira district is the most vulnerable (13). In this upazilla, due to their geographic location and position, the five union namely Buri Goalini, Gabura, Munshiganj, Ramjan Nagar, and Kaikhali union are the main victims of climate change (Figure 2).

Women are the more vulnerable population in climatic issues as compared to men (14). Considering these issues, this study has delved into women’s health issues and these locations for getting climatic issues. Women in reproductive ages are the most vulnerable among women of different age groups. This study employed multi-method approach combining in-depth interview (IDI) and Key Informants Interviews (KIIs). The health situations or problems of women were recorded through IDI, and their stated narratives were then complemented with the voices of KIIs. The primary participants of the study were the women aged between 14 and 49 (age at menarche and age at menopause). This is the average age group of women in reproductive age in the context of Bangladesh. Other attributes, such as marital status, or having children or not, have not been considered assuming that these attributes could have minimal influences. However, this study took respondents from different age cohorts, i.e., 14–20, 21–30, 31–40, and 41–49 to ensure diversified health experiences along with their aging and socio-economic experiences. Different occupational statuses have also been taken into consideration to get a flavor of different socio-economic communities. Besides to get insight into the health complexities of women, 05 married male individuals were taken for IDI. They have been asked about their observation of health complexities of women, a condition created through climate changes and associated health care facilities (Table 3).

**Table 3:** Demographic profile of the respondents of IDI.

S. L.	Name	Gender	Age	Union (location)	Profession
1	Ayesha Khatun	Female	38	Buri Goalini	Housewife
2	Marufa Begum	Female	15	Buri Goalini	Student
3	Jahanara Akter	Female	27	Buri Goalini	Tailor
4	Sufia Begum	Female	44	Buri Goalini	Reared duck & hen
5	Rasheda Parvin	Female	33	Buri Goalini	Worked in shrimp cultivation
6	Salma Akter	Female	29	Gabura	Housewife
7	Tamanna Sultana	Female	18	Gabura	Student
8	Nurjahan Begum	Female	36	Gabura	Agriculture
9	Rozina Khatun	Female	45	Gabura	Housewife
10	Shahana Yesmin	Female	31	Gabura	Tailor
11	Selina Begum	Female	39	Munshiganj	Housewife
12	Asma Ara	Female	25	Munshiganj	Engaged in a job
13	Jesmin Khatun	Female	28	Munshiganj	Agriculture
14	Momtaz Begum	Female	47	Munshiganj	Housewife
15	Khadija Akter	Female	20	Munshiganj	Student
16	Rahima Begum	Female	42	Ramjan Nagar	Housewife
17	Ferdousi Ara	Female	30	Ramjan Nagar	Housewife
18	Fatema Sultana	Female	24	Ramjan Nagar	Student
19	Amena Khatun	Female	37	Ramjan Nagar	Rearing duck & hen
20	Nasima Begum	Female	32	Ramjan Nagar	Engaged in a job
21	Mahmuda Khatun	Female	35	Kaikhali	Housewife
22	Hasina Begum	Female	40	Kaikhali	Agriculture
23	Mitu Akter	Female	19	Kaikhali	Student
24	Rowshan Ara	Female	28	Kaikhali	Housewife
25	Lucky Begum	Female	27	Kaikhali	Housewife
26	Md. Abdul Karim	Male	31	Kaikhali	Unemployed
27	Md. Rashid Mia	Male	34	Munshiganj	Worked in shrimp cultivation
28	Md. Shahidul Islam	Male	48	Ramjan Nagar	Govt. Service
29	Md. Jamal Uddin	Male	53	Gabura	Ex-Govt. job
30	Md. Kamrul Islam	Male	41	Buri Goalini	Agriculture

The purpose of adopting KIIs was to identify and check the validity of the understanding of local people about the impact. Also, this group has produced generous comments on the existing water availability, institutional functionalities and healthcare facilities. In this group, we have interviewed community-level health care workers who are locally and institutionally called Community Health Care Provider (CHCP), Health Inspector (HI), Family Welfare Assistant (FWA), Upazila (sub-district) level medical officer, district-level doctors, and other associated health care providers. All of these groups are mostly associated and key informants who share their insights on the health issues of women induced by climatic incidents.

### 2.1 Ethical considerations

Ethical considerations are crucial as it centers on women of reproductive age in at-risk coastal communities. Participants were provided detailed information regarding the study’s objectives, methodologies, and any possible risks or advantages to facilitate informed consent. Participation was completely voluntary, allowing subjects to leave the study at any moment without incurring any adverse repercussions. The privacy and confidentiality of participants were protected by anonymizing identifiable information and securely archiving all data, allowing access solely to approved members of the research team. Special consideration was afforded to the cultural and gender-specific sensitivities of the participants to ensure their dignity and comfort were upheld during the research process. Measures were implemented to mitigate harm by fostering a supportive environment during data gathering, especially considering the sensitive nature of the subjects addressed. When distress occurred, participants were provided with access to suitable professional support options. The research complied with international ethical principles and obtained prior clearance from pertinent institutional review boards to ensure adherence to ethical standards throughout the investigation.

## 3 Results and discussion

### 3.1 Availability of fresh water and nature of water consumption

Access to potable water near residential areas is extremely limited. The upper surface salinity affects shallow tube wells, leaving them to supply mostly saline water. Communities rely on privately owned deep-water plants located an average of 3–5 kilometers away. Women are primarily responsible for water collection. Families, including children and pregnant women, ration water carefully. Pregnant women often lack sufficient water, leading to health issues: “We count every drop of water to use it wisely. Sometimes, we endure thirst because there is not enough fresh drinking water. In extreme cases, we resort to drinking pond water, which we try to boil thoroughly, but it still tastes salty” (Ayesha Khatun, Female, 38, Buri Goalini, Housewife). Sometimes they run out of water: “sometimes at night we run out of water. We ask our neighbor for drinking water. We help each other in emergency situations like this” (Marufa Begum, Female, 15, Buri Goalini, Student). When money is unavailable, families fetch water from ponds or nearby sources, even during emergencies. During summer, there creates extra demand for water due to the harsh climate: “During summer, every day, I drink at least 5 liters of water … otherwise I cannot survive due to the hard work in the field” (Md. Kamrul Islam, Male, 41, Buri Goalini, Agriculture). Moreover, ponds dry up, further limiting water access: “in summer, all the ponds dry up, and we cannot even get fresh water for bathing” (Md. Abdul Karim, Male, 31, Kaikhali, Unemployed). In some areas local government authorities introduced piped- based fresh water supply systems while a major portion of the community members could hardly afford them. “Recently … we will have to pay 150 taka a month even if we do not use the piped water” (Rasheda Parvin, Female, 33, Buri Goalini, Worked in shrimp cultivation). Many individuals do not drink enough water daily and show limited awareness of dehydration’s risks, such as dryness, dark urine, and headaches. Knowledge about causes of high blood pressure (stress, genetics, water salinity, extreme heat) is also lacking. Similarly, respondents often do not know how to properly treat pond or rainwater (e.g., boiling, using bleaching powder, or Fitkari- alum or potassium alum) or why keeping rainwater clean is crucial to prevent diarrhea, skin infections, and stomach problems. Moreover, there are significant gaps in understanding basic hygiene, safe water practices, and proper waste disposal to avoid illnesses like diarrhea.

### 3.2 Use of saline water and water-borne health issues

Saline water is used for nearly all household tasks, including washing faces, brushing teeth, performing ablution, washing clothes, cleaning houses, cooking, and feeding livestock. Its usage, though harmful, is unavoidable, even during pregnancies or illnesses: “we cannot wash our faces because the saline water burns our eyes, but we have no other choices. During summer, our skin burns when we touch it” (Salma Akter, Female, 29, Gabura, Housewife).

Pond water, though slightly less saline, is also used for bathing, washing clothes, and ablution. However, the number of protected ponds, which prevent salinity intrusion, is minimal: “Men can go to distant ponds to bathe, but women must use saline water, which causes itching” (Md. Rashid Mia, Male, 34, Munshiganj, Work in shrimp cultivation). Saline water also damages the soil, reducing agricultural productivity: “Vegetables and fruits barely grow because of climate problems. Salinity in the soil prevents regular vegetable cultivation” (Jesmin Khatun, Female, 28, Munshiganj, Agriculture). Doctors report that prolonged use of saline water causes numerous physical and mental health issues, including skin diseases such as itching, dry skin, eczema, fungal infections, and rashes. “Most of us suffer from itching, and in summer it becomes unbearable” (CHCP, Male, Gabura). Chronic conditions often develop as early symptoms are neglected. Diarrhea is widespread, affecting individuals for about 10 days each month (15). “Diarrhea and dysentery are so common that we do not care … we buy medicine from the grocery store as per our needs” (Md. Jamal Uddin, Male, 53, Gabura, Ex-Govt. job). Women and adolescent girls face additional challenges such as white discharge, high blood pressure, gastritis, vaginal itching, abdominal pain, and diarrhea. Long-term consumption of saline water contributes to breast and vaginal cancers. Common problems include dysmenorrhea (painful periods) and leukocoria. Pregnant women frequently experience preeclampsia, dizziness, malnutrition, anemia, and premature abortions. Skin brightness diminishes from frequent exposure to saline water, particularly in children aged 7–8 and older. Other common issues include dry skin, premature aging, wrinkling, hair loss, and white or gray hair during summer due to imbalances in melanin and tocopherol. “During summer, the color of our hair and skin gets changed, and the skin burns because of the extreme salinity” (Rozina Khatun, Female, 45, Gabura, Housewife). Alkaline saline water irritates the eyes, leading to burning sensations, and causes nail decay and discoloration of teeth. “Eye burning is unbearable due to saline water while washing the face” (Tamanna Sultana, Female, 18, Gabura, Student).

Many women are unaware that prolonged itching, redness, dryness, or rashes may require medical attention. Most recognize a connection between salty water and skin issues, yet knowledge of essential preventive measures—like using fresh water and avoiding sun exposure—remains limited. While a few rely on herbal remedies or do nothing, many turn to self-medication without professional guidance. “People do not come to us for issues like hair loss or skin brightness loss because they have accepted these as normal” (Medical Officer, Upazila Hospital).

### 3.3 Challenges during heat waves

Heat waves exacerbate difficulties, with climate change making extreme weather more common while most of the community members could hardly recognize it: “We do not know the term ‘heat wave’ or ‘heat stroke’” (Ferdousi Ara, Female, 30, Ramjan Nagar, Housewife). They continue their normal activities without concerning the heat related health difficulties: “Often time we get unconscious from extreme heat” (Nasima Begum, Female, 32, Ramjan Nagar, Engaged in a job). Common heat-related illnesses include diarrhea, dysentery, vomiting, heat stroke, dehydration, and high blood pressure. Farmers and laborers report sudden deaths during extreme heat: “In our village, a farmer died while working in the field for extreme heat” (Md. Shahidul Islam, Male, 48, Ramjan Nagar, Govt. Service). Electricity shortages further worsen the situation, with only 5–6 h of power daily: “Load-shedding worsens the situation as electricity is available only for 5–6 h a day on average.” (Amena Khatun, Female, 37, Ramjan Nagar, Rearing duck & hen). Pregnant women suffer from conditions such as gastritis, high blood pressure, and blurred vision during heat waves. These conditions often lead to preterm deliveries and an increased need for C-sections: “A woman became severely ill with diarrhea during a heat wave and had to be admitted to a hospital far away because no local facilities could provide adequate care.” (Nurse, Shyamnagar Upazila Health Complex). During extreme heat waves, dehydration, blurred vision, dizziness, heat stroke, and vomiting are common. A respondent noted as “I work in a brick kiln. During heat wave I have blurry vision, dizziness and dehydration.” (Rahima Begum, Female, 42, Ramjan Nagar, Housewife). Saline water consumption worsens hypertension and chronic conditions like irritable bowel syndrome (IBS). Due to widespread poverty, people rely on herbal and traditional remedies for health problems. The saline environment inhibits farming, leaving vegetables and poultry scarce. Malnourishment is prevalent, and protein demands are unmet. “Prescribing medicine alone will not solve their problems. The root cause lies in their poor socio-economic conditions.” (CHCP, Female, Gabura Union Community Clinic). Many women have limited awareness of extreme heat symptoms like headaches, dizziness, high body temperature, or reduced sweating. Most are also unfamiliar with causes such as high humidity, lack of cooling spaces, and dehydration. While some know to rest in a cool area or wear lightweight clothing, many do not realize the importance of drinking adequate water or using wet cloths to cool down.

### 3.4 Menstrual blood management and related health issues

Many women rely on unhygienic materials to manage menstrual blood, with only a small portion using sanitary napkins or clean cloth. Women who use sanitary napkins face difficulties in used-pad management and disposals: “Most of us use sanitary pads but we face difficulties to dump them especially when we are at school” (Fatema Sultana, Female, 24, Ramjan Nagar, Student). Many women use cloth instead of sanitary napkins, washing and drying them in unhygienic conditions, which leads to infections: “We use old clothes again and again without washing them properly … germs are not properly reduced, and we get infected.” (Rowshan Ara, Female, 28, Kaikhali, Housewife). Unsafe cleaning and drying practices, such as using contaminated water or keeping garments covered and away from sunlight, further elevate health risks. “They prefer to hide their used cloth during menstruation due to shyness; that is why they dry these cloths an unhealthy way, not in sunlight.” (Lucky Begum, Female, 27, Kaikhali, Housewife). Cultural beliefs often restrict certain foods and activities during menstruation, though most do not follow taboos related to carrying a newborn or touching item in a deceased person’s home: “We do not have enough education on menstruation … No community clinic worker visits us to provide the knowledge” (Mitu Akter, Female, 19, Kaikhali, Student). Menstrual pain is frequently left untreated or managed through traditional methods, with few seeking professional care. Menstruation-related problems are widespread among women due to poor hygiene and limited access to medical care. Shyness and taboos prevent open discussions about menstrual health: “Talking about menstruation openly is considered taboo here.” (Hasina Begum, Female, 40, Kaikhali, Agriculture). Although community clinics provide free iron, folic acid, and calcium tablets, sanitary napkins are not available.

### 3.5 Vector-borne disease: dengue

Changing climate conditions—such as rising temperatures, irregular rainfall, and saltwater intrusion—are intensifying the risk of dengue transmission (16). Higher temperatures and unpredictable monsoon patterns create ideal breeding grounds for mosquitoes (17), while saline water sources limit access to safe drinking water and hygiene practices. In this environment, many women already face challenges in recognizing and managing dengue, as knowledge of its full range of symptoms and appropriate healthcare measures remains incomplete: “People are not aware of dengue.” (Momtaz Begum, Female, 47, Munshiganj, Housewife). During our interview, most women recognize that dengue is transmitted through mosquito bites, though a smaller group remains unaware of its cause. High fever is commonly identified as a key symptom, but knowledge about other signs—such as severe headaches, vomiting, body pain, or rashes—is limited. While many know they should visit a health center if infected, fewer understand the importance of rest, hydration, or using mosquito nets. Half are informed about nets and regularly disposing of stagnant water to prevent mosquito breeding, but most do not realize that covering the body can also help avoid bites: “Half of us know to use nets and get rid of stagnant water, but most do not realize covering the body also helps avoid bites” (Jesmin Khatun, Female, 28, Munshiganj, Agriculture). Additionally, the strain of climate change on local livelihoods can limit resources for preventive measures like mosquito nets, repellents, or regular disposal of standing water. “The health complex cannot cater to dengue patients as it lacks the necessary facilities.”—Midwife, Shyamnagar Upazila Health Complex. Greater community awareness and adaptation strategies are crucial: ensuring reliable freshwater supplies, promoting proper waste management, and educating communities about the importance of rest, hydration, and timely medical attention can significantly reduce dengue risks.

### 3.6 Health issues during pregnancy

Many pregnant women in the coastal region of Satkhira have partial knowledge about proper nutrition, often unaware of the need to eat enough staple foods or to avoid uncooked meats. Although many recognize that insufficient nutrition can harm both mother and baby, awareness of specific risks, necessary checkups, and danger signs remains limited. Stress, inadequate diets, and hard physical labor are not widely seen as factors affecting maternal health. While some understand the importance of rest, regular medical visits, and staying hydrated, most do not realize how critical these measures are in preventing complications. Pregnant women frequently experience anemia, dizziness, malnutrition, and high blood pressure. Early marriage and malnutrition contribute to a high rate of C-sections. Climate change limited their daily income earnings: “We could hardly manage work during the monsoon period. Extreme heat exposure limits us to stay home. Besides natural calamities, e.g., cyclone, flooding, excessive rainfall add extra burden in our daily livings” (Md. Abdul Karim, Male, 31, Kaikhali, Unemployed). Within these circumstances, most of the household set limit on their daily meals, prioritizing pregnant women so as they could provisioned with nutrition: “We cut our meals to feed our pregnant women but still they remain malnourished.” (Asma Ara, Female, 25, Munshiganj, Engaged in a job). As women are the main duty bearer of collecting water, during pregnancy they could not perform this duty which lead extra household expenditure for fresh water collection: “As our women cannot carry drinking water during pregnancy, we have to take the van delivery service to collect water.” (Khadija Akter, Female, 20, Munshiganj, Student). Many women avoid seeking prenatal care due to financial and logistical barriers. Community clinics promote regular check-ups and provide nutritional advice, including local foods rich in folic acid, iron, and calcium, such as taro stems, bananas, and small fish. Despite efforts, preterm deliveries remain common: “We advise pregnant women to take necessary supplements and provide personal contact numbers for emergencies.” (FWA, Munishiganj Union, Female). Using saline water increases vaginal pH levels, leading to infections and complications like cervicitis and chorioamnionitis. This often necessitates C-sections. Addressing these health issues through community clinics and improving access to fresh water is critical. Gynecological issues are prevalent during pregnancy due to the use of saline water, the lack of community clinics, and insufficient post-C-section care. Lack or scarcity of fresh water due to climatic change creates a burden for women in terms of sufficient water consumption during pregnancy: “Our pregnant women do not get enough drinking water as the water is limited.” (Selina Begum, Female, 39, Munshiganj, Housewife). Many women resort to using ointments for itching, as they have no female health workers to confide in. The rate of C-sections is high, often causing financial strain for families. “The prevalence of C-sections is significant, and tensions often arise due to financial constraints.”—Medical Officers, Shyamnagar Upazila Health Complex (7).

### 3.7 Hygiene and treatment behavior

Hygiene and sanitation remain critical but challenging due to poorly constructed latrines and inadequate equipment. Many residents lack interest in improving their sanitation standards referring frequent occurrence of disasters: “Every year cyclone destroys our outdoor house structure. Flooding caused overloading safety faucal tank. In that situation what is the meaning to invest on a highly equipped sanitation structure? (Md. Shahidul Islam, Male, 48, Ramjan Nagar, Govt. Service). Residents often rely on traditional medicine or over-the-counter drugs from local shops for common ailments like diarrhea and skin diseases, bypassing doctors altogether: “Rather than talking about the disease with the health workers, they prefer to take only medicine for that disease.” (Md. Abdul Karim, Male, 31, Kaikhali, Unemployed). This practice frequently results in chronic conditions due to improper treatment. “We do not visit doctors for diarrhea or dysentery; we just buy medicine from the grocery shop” (Mahmuda Khatun, Female, 35, Kaikhali, Housewife). Many also turn to quacks or village doctors due to the distance to formal health complexes. Traditional treatments are prevalent, with residents believing they have adequate knowledge about common ailments: “These diseases are so common that we all know the medicines for them, so there’s no need to visit a doctor” (Md. Kamrul Islam, Male, 41, Buri Goalini, Agriculture).

### 3.8 Mental health conditions

The mental health of community members is heavily impacted by the daily struggle to fetch water, often leading to stress and sleeplessness: “I have to take medicine to sleep.” (Lucky Begum, Female, 27, Kaikhali, Housewife). Many women are aware that mood changes can happen during or after pregnancy, but fewer recognize that more serious emotional challenges—like depression or loneliness—may also arise. Knowledge about specific warning signs, such as changes in sleep patterns or feelings of guilt, is limited, and many do not know simple coping strategies like rest, light exercise, healthy eating, or seeking social support. “If my wife collects water, we save on transportation costs, but it becomes an extra burden for her. She gets angry if I stay home and do not help.” (Md. Jamal Uddin, Male, 53, Gabura, Ex-Govt. job). Financial pressures and disputes over borrowed money also contribute to mental health challenges: “I cannot sleep at night as I am always worried about the installment fees.” (Md. Abdul Karim, Male, 31, Kaikhali, Unemployed). Women often experience irritability during menstruation, further exacerbated by the stress of collecting water from remote locations.

### 3.9 Linking the existing situation with climatic issues

**Figure 3:**
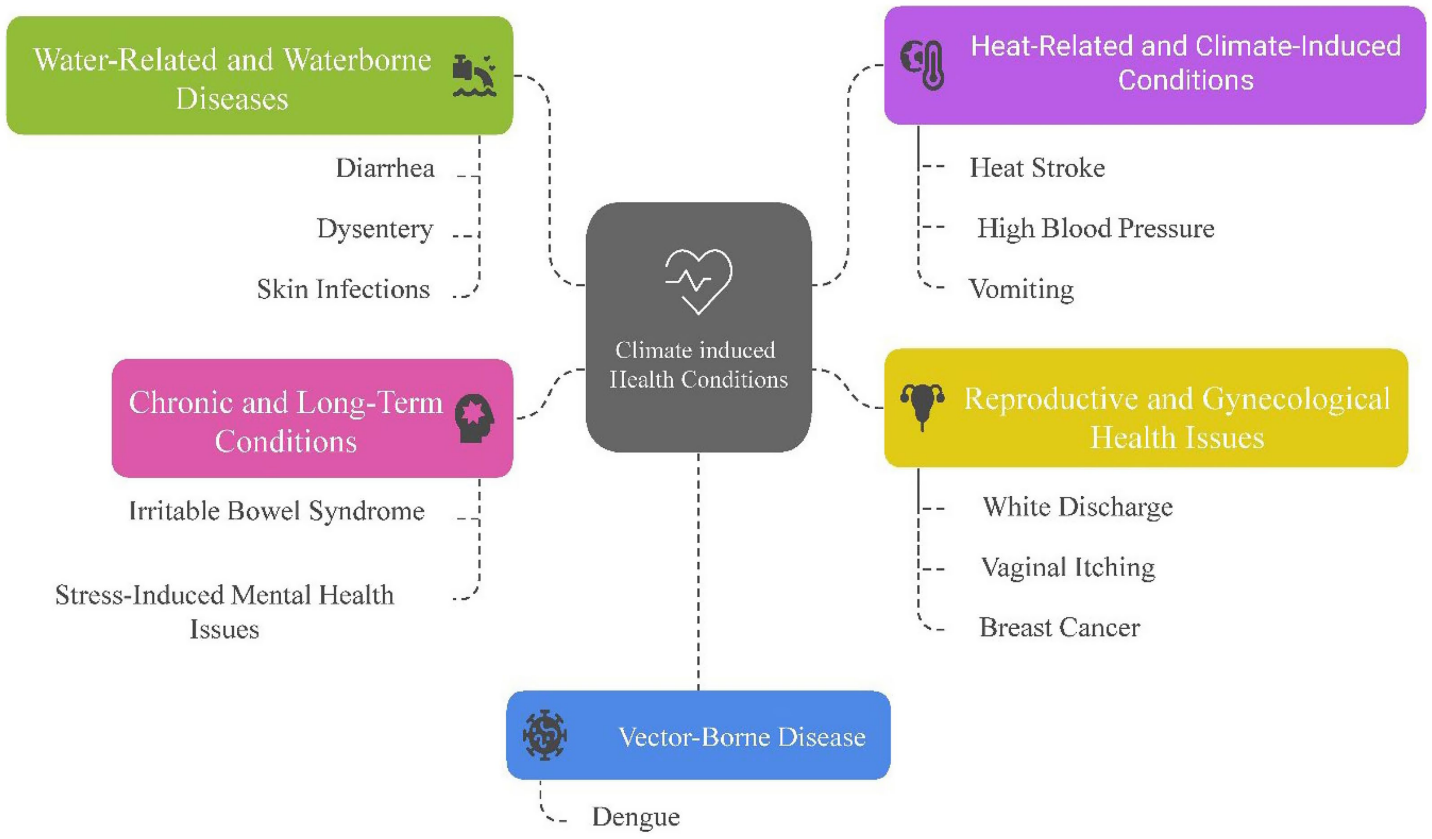
Prevalence of identified frequent climate induced health problems. Source: Compiled by authors.

The health challenges faced by the communities are intricately tied to the ongoing impacts of climate change. Rising sea levels and saltwater intrusion have drastically reduced access to fresh water, forcing families to rely on saline water for daily needs. This has led to a cascade of health problems, including dehydration, skin diseases, and gastrointestinal infections, with chronic conditions like high blood pressure becoming alarmingly common. Pregnant women are particularly vulnerable, enduring complications such as anemia (18), preeclampsia (19), and even premature births due to insufficient water and nutrition. Pregnant women often require urgent care for complications that local healthcare systems may be ill-equipped to handle (20). Heat waves, intensified by climate change, exacerbate these struggles. The extreme heat not only increases dehydration and heat strokes but also compounds existing health problems. Pregnant women frequently face complications requiring urgent care, which is often unavailable locally. The unrelenting heat also amplifies mental health issues, as the burden of collecting water and managing household tasks weighs heavily on women, leading to stress and emotional strain. The region’s vulnerability extends to vector-borne diseases like dengue, as erratic rainfall and warmer temperatures create ideal mosquito breeding conditions. Limited awareness and inadequate preventive measures leave communities ill-equipped to handle outbreaks, further straining their already fragile healthcare system (21) (Figure 3).

**Figure 4:**
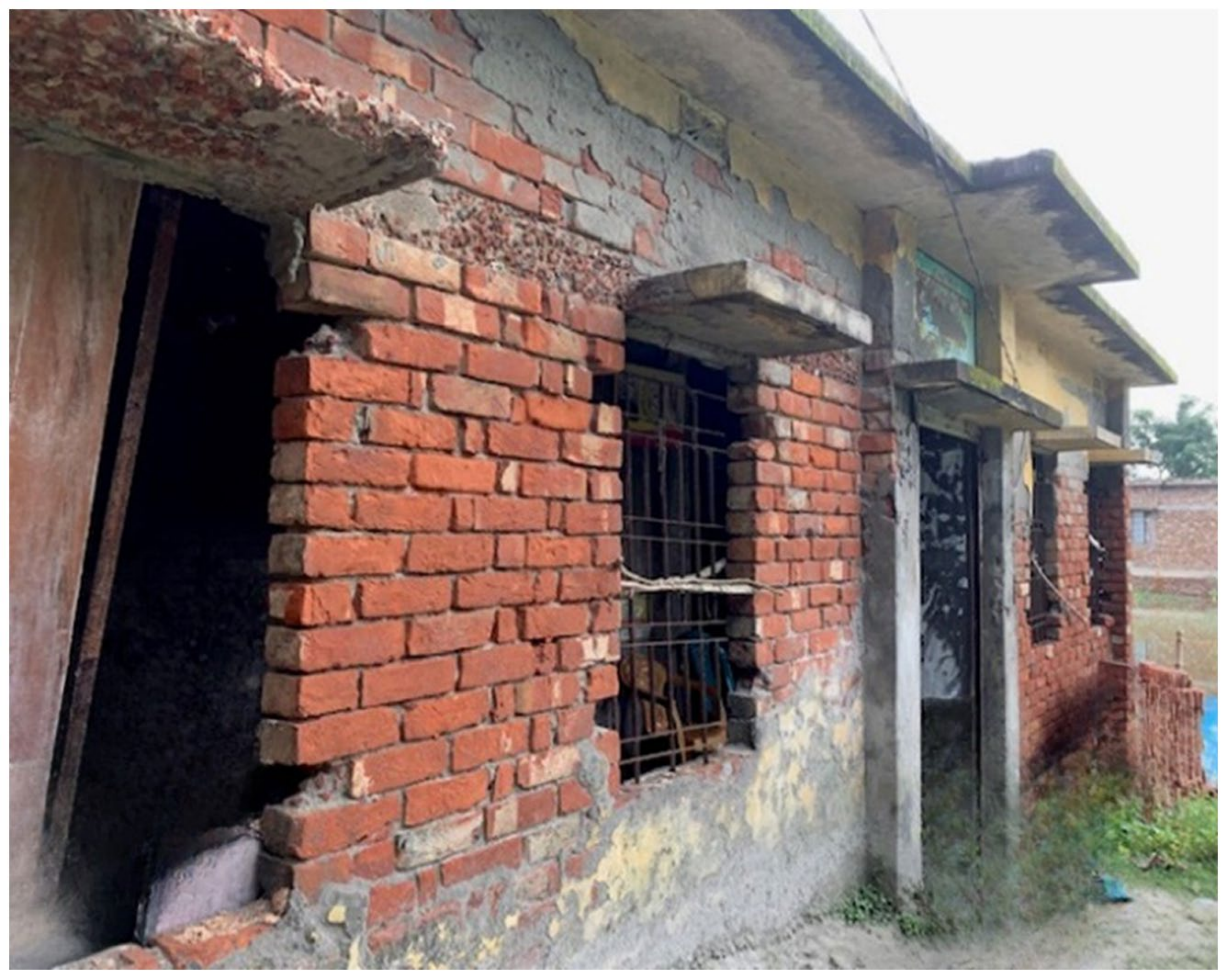
Salinity affecting the local healthcare center (Community Clinic). Source: Authors during field work.

**Figure 5:**
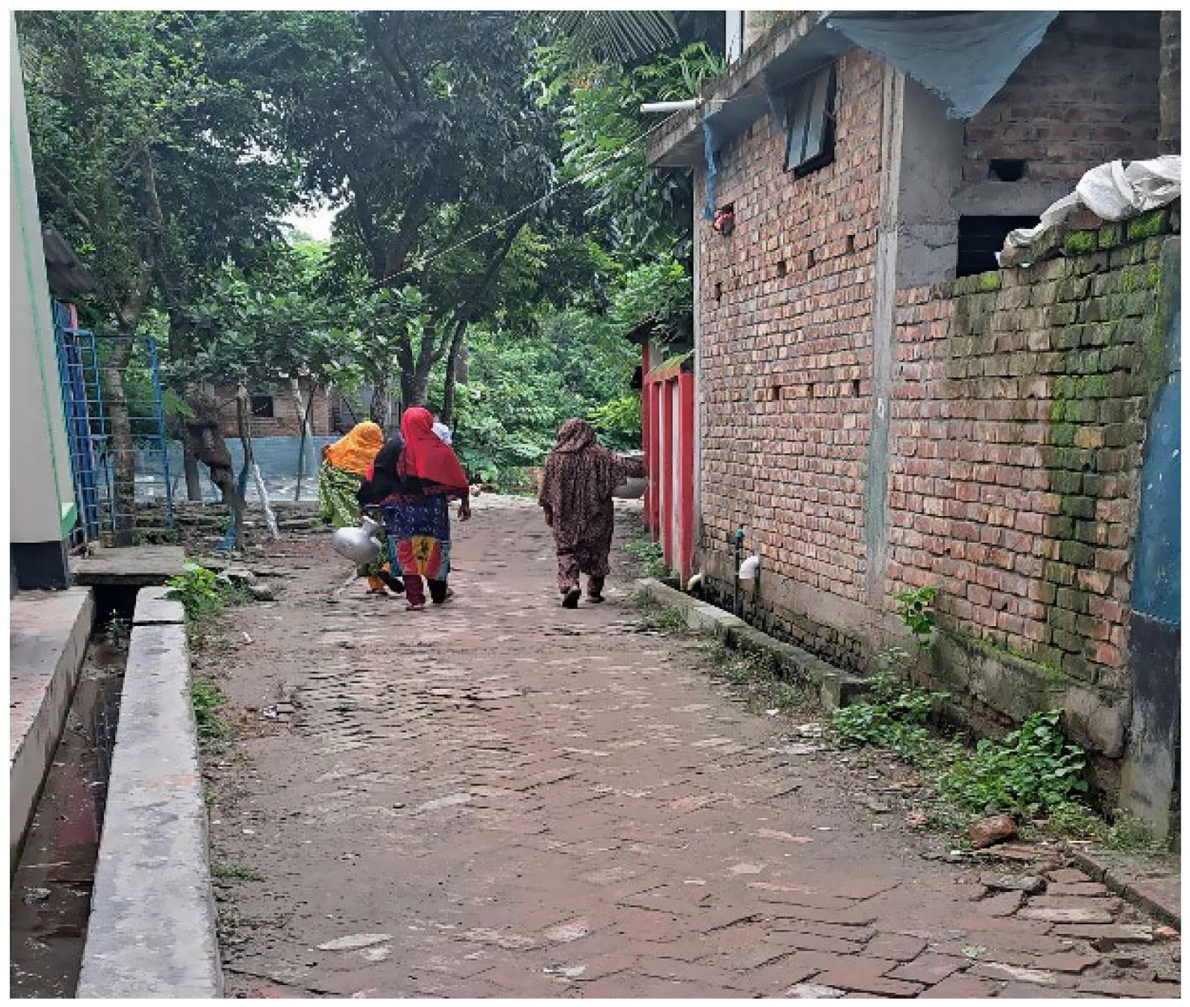
Women on the way to their regular activities, i.e., fresh water collection. Source: Authors during field work.

Hygiene and sanitation challenges, rooted in the lack of clean water, also take a toll. Women struggle with menstrual health due to inadequate facilities and cultural taboos, leading to infections and untreated illnesses (22). Meanwhile, saline water corrodes not just the health of individuals but also the fertility of the soil, reducing agricultural output and pushing families toward malnutrition and food insecurity (23) (Figures 4, 5).

The pervasive effects of climate change have seeped into every aspect of life, turning daily survival into a constant struggle. Beyond physical health, the psychological burden of coping with these challenges, particularly for women, is immense. Stress, anxiety, and a sense of helplessness pervade the community, fueled by poverty and the relentless demands of an unforgiving environment (24). In this narrative of intertwined vulnerabilities, climate change acts as the catalyst, amplifying existing disparities and creating new challenges. Addressing these issues requires not just immediate interventions for water and healthcare access but also long-term strategies for climate resilience and community empowerment (25). Only by tackling these challenges holistically can we hope to break this cycle of vulnerability and create a healthier, more sustainable future.

### 3.10 Suggested strategies to improve health care services

Community voices highlight that water and health investments must move together: households cannot practice hygiene or utilize clinic services if fresh water is unavailable, while clinics cannot deliver safe maternity care without reliable water, medicines and trained (especially female) staff. The integrated package above therefore addresses both supply-side and demand-side gaps identified throughout the study.

### 3.11 Strategies for safer, more reliable water

**Table:** 

Focus	Community-proposed action (speaker in-text)
Piped / deep-tube supply	“Government needs to take initiatives to provide fresh drinking water … and accelerate the BWDB master water-plant project” (Ayesha Khatun, Female, 38, Buri Goalini, Housewife)
Village purification hubs	“Water-purifying systems should exist in at least two points of every village and be run under government ownership so users pay the lowest cost” (Rasheda Parvin, Female, 33, Buri Goalini, Worked in shrimp cultivation)
Salinity-proof ponds	“If ponds can be bounded highly, then the water can be used without salinity” (Sufia Begum, Female, 44, Buri Goalini, Reared duck & hen)
Rain-water harvesting	“Coordinate with private pond owners to harvest rainwater for public use and train households in rain-water-harvesting methods” (Md. Kamrul Islam, Male, 41, Buri Goalini, Agriculture)
More sources & awareness	“Increase the total number of fresh-water sources in the area and raise awareness on correct drinking-water practices” (Salma Akter, Female, 29, Gabura, Housewife)
Water for health facilities	“Different stakeholders should come forward to solve the fresh-water shortage at the Upazila Health Complex” (Tamanna Sultana, Female, 18, Gabura, Student)

### 3.12 Strategies to strengthen local health services

**Table:** 

Focus	Community-proposed action (speaker in-text)
Female staff & privacy	“Increase the number of female health workers … employ female doctors so women can share gynecological problems without shame” (Selina Begum, Female, 39, Munshiganj, Housewife)
Active community clinics	“Regulate community clinics properly; announce outreach sessions by miking so illiterate residents attend” (Shahana Yesmin, Female, 31, Gabura, Tailor)
Essential drugs & kit	“Shortage of necessary medical equipment must be fixed as soon as possible” (Md. Jamal Uddin, Male, 53, Gabura, Ex-Govt. job)
Dedicated maternity care	“Build a child-delivery center with emergency equipment, doctors and nurses; introduce an ambulance/van service for pregnant women” (Asma Ara, Female, 25, Munshiganj, Engaged in a job)
Clean water inside clinics	“Provide a clean-water delivery system for the health complex; dirty pond water is unusable when ponds dry out” (Md. Shahidul Islam, Male, 48, Ramjan Nagar, Govt. Service)
Home-based ANC / PNC	“Clinic workers should visit pregnant women every 28 days and advise hospital check-ups every 1–2 months” (Khadija Akter, Female, 20, Munshiganj, Student)
Cost relief (DSF cards)	“Expand DSF cards so marginalized pregnant women get free or subsidized care” (Nasima Begum, Female, 32, Ramjan Nagar, Engaged in a job)
Menstrual-hygiene support	“Coordinate with the Health Ministry to supply sanitary pads—even on a small scale—and hold yard meetings on safe disposal” (Lucky Begum, Female, 27, Kaikhali, Housewife)
Behavior-change education	“Build awareness of consulting doctors before buying medicines” and run heat-wave/dengue sessions on rest, hydration, nets and waste management (Mahmuda Khatun, Female, 35, Kaikhali, Housewife)

## 4 Conclusion

This study highlights the profound and multifaceted health impacts arising from climate change in vulnerable coastal communities. The interconnections between environmental changes—such as rising sea levels, saltwater intrusion, and erratic weather patterns—and critical health challenges underscore the urgency of addressing these issues in an integrated manner. Water scarcity and salinity, exacerbated by climate change, have become central barriers to health and well-being, leading to widespread dehydration, malnutrition, and waterborne diseases. The inadequate access to safe water and sanitation amplifies risks for women, children, and pregnant mothers, compounding vulnerabilities with long-term consequences for physical and mental health. Climate-induced agricultural decline further worsens food insecurity and malnutrition, creating a cascading effect on overall health. Heat waves and the increasing prevalence of vector-borne diseases like dengue illustrate how climate change intensifies existing public health threats, stretching already limited healthcare resources. The disproportionate impact on women, particularly regarding menstrual hygiene and maternal health, highlights the urgent need for gender-sensitive solutions. Moreover, the mental health burden resulting from these compounding challenges emphasizes the hidden psychological toll of climate change on affected populations.

Addressing these challenges requires a holistic approach that integrates climate resilience with improved access to essential services. Priority actions include ensuring a reliable supply of fresh water, promoting climate-resilient agriculture, strengthening healthcare systems, enhancing community awareness of hygiene and disease prevention, and providing targeted support for women and vulnerable groups. This study serves as a call to action for policymakers, development practitioners, and community leaders to prioritize adaptive strategies and investments that address both the environmental and health impacts of climate change. By empowering communities with the climate adaptive tools and provisioning climatic health impact knowledge to navigate these challenges, it is possible to build a foundation for sustainable health and resilience in the face of a changing climate.

## Data Availability

The raw data supporting the conclusions of this article will be made available by the authors, without undue reservation.
